# The Registrar Clinical Encounters in Training (ReCEnT) cohort study: updated protocol

**DOI:** 10.1186/s12875-022-01920-7

**Published:** 2022-12-16

**Authors:** Andrew Davey, Amanda Tapley, Mieke van Driel, Elizabeth Holliday, Alison Fielding, Jean Ball, Katie Mulquiney, Katie Fisher, Neil Spike, Lisa Clarke, Dominica Moad, Anna Ralston, Irena Patsan, Benjamin Mundy, Alexandria Turner, Jordan Tait, Lucrezia Tuccitto, Sarah Roberts, Parker Magin

**Affiliations:** 1 School of Medicine and Public Health, The University of Newcastle, University Dr, Callaghan, NSW 2308 Australia; 2 NSW & ACT Research and Evaluation Unit, GP Synergy, Level 1, 20 McIntosh Dr, Mayfield West, NSW 2304 Australia; 3 Faculty of Medicine, General Practice Clinical Unit, The University of Queensland, 288 Herston Road, Brisbane, QLD 4006 Australia; 4 Clinical Research Design and Statistical Support Unit (CReDITSS), Hunter Medical Research Institute (HMRI), Lot 1, Kookaburra Cct, New Lambton Heights, NSW 2305 Australia; 5 Eastern Victoria General Practice Training (EVGPT), 15 Cato Street, Hawthorn, VIC 3122 Australia; 6 Department of General Practice and Primary Health Care, University of Melbourne, 200 Berkeley Street, Carlton, VIC 3053 Australia; 7 Faculty of Medicine, Nursing and Health Sciences, School of Rural Health, Monash University, Churchill, VIC 3842 Australia; 8 General Practice Training Tasmania (GPTT), Level 3, RACT House, 179 Murray Street, Hobart, TAS 7000 Australia

**Keywords:** General Practice, Family Practice, Education, Medical, Graduate, Epidemiology, Physician–Patient Relations

## Abstract

**Background:**

During vocational general practice training, the content of each trainee’s (in Australia, registrars’) in-consultation clinical experience is expected to entail a breadth of conditions that exemplify general practice, enabling registrars to gain competency in managing common clinical conditions and common clinical scenarios. Prior to the Registrar Clinical Encounters in Training (ReCEnT) project there was little research into the content of registrars’ consultations despite its importance to quality of training. ReCEnT aims to document the consultation-based clinical and educational experiences of individual Australian registrars.

**Methods:**

ReCEnT is an inception cohort study. It is comprised of closely interrelated research and educational components. Registrars are recruited by participating general practice regional training organisations. They provide demographic information about themselves, their skills, and their previous training. In each of three 6-month long general practice training terms they provide data about the practice where they work and collect data from 60 consecutive patient encounters using an online portal. Analysis of data uses standard techniques including linear and logistic regression modelling. The ReCEnT project has approval from the University of Newcastle Human Research Ethics Committee, Reference H-2009–0323.

**Discussion:**

Strengths of the study are the granular detail of clinical practice relating to patient demographics, presenting problems/diagnoses, medication decisions, investigations requested, referrals made, procedures undertaken, follow-up arranged, learning goals generated, and in-consultation help sought; the linking of the above variables to the presenting problems/diagnoses to which they pertain; and a very high response rate. The study is limited by not having information regarding severity of illness, medical history of the patient, full medication regimens, or patient compliance to clinical decisions made at the consultation. Data is analysed using standard techniques to answer research questions that can be categorised as: mapping analyses of clinical exposure; exploratory analyses of associations of clinical exposure; mapping and exploratory analyses of educational actions; mapping and exploratory analyses of other outcomes; longitudinal ‘within-registrar’ analyses; longitudinal ‘within-program’ analyses; testing efficacy of educational interventions; and analyses of ReCEnT data together with data from other sources. The study enables identification of training needs and translation of subsequent evidence-based educational innovations into specialist training of general practitioners.

**Supplementary Information:**

The online version contains supplementary material available at 10.1186/s12875-022-01920-7.

## Background

Australian general practice registrars (post-graduate trainees in specialist general practice), like family medicine/general practice vocational trainees in many other countries, train within an apprenticeship-like model [1].Registrars’ training (including their clinical placements) is organized and administered by geographically-defined, not-for-profit educational organizations (currently (2016–2022) Regional Training Organisations (RTOs) and, previously (2010–2015), Regional Training Providers (RTPs)) within a national program [1, 2]. While RTOs/RTPs deliver regular away-from-practice education sessions, the majority of registrars’ learning occurs in-practice, within an apprenticeship-like model. Within this model, as in post-graduate medical training generally [3], consulting with patients is the core learning activity – the ‘curriculum walks in the door’ [1].

During training, the content of each registrar’s in-consultation clinical experience is expected to encompass “common and significant conditions that exemplify general practice” [4] and to enable registrars to gain competency in managing common clinical conditions and common clinical scenarios [5–7]. The rationale is that the development of sound clinical skills requires exposure to ‘an adequate database’ of clinical cases to facilitate ‘non-analytic’ diagnostic reasoning skills [8]. Adequate ‘patient mix’ has been found to be associated with self-reported learning outcomes, including the experienced quality of the learning program [9]. In the Australian apprenticeship-like training model, however, the patient demographic profile and range of clinical conditions encountered varies, sometimes markedly, between individual practices. This may compromise the adequacy of a registrar’s in-training clinical exposures.

The context of this in-consultation learning is that, though registrars have considerable clinical autonomy (prescribing, test-ordering, referral, and billing rights equivalent to established general practitioners (GPs)), they have recourse to advice and assistance from an assigned, RTO-accredited GP supervisor (or the supervisor’s delegate). The supervisor also delivers regular dedicated, one-on-one, in-practice educational sessions.

While the adequacy of the breadth of registrars’ in-consultation experience is of considerable importance, prior to the Registrar Clinical Encounters in Training (ReCEnT) project there was little research into the content of registrars’/trainees’ consultations. There has been, however, systematic and in-depth examination of the clinical experience of established GPs/primary care physicians [10], notably in the Primary Care Network Survey and the National Ambulatory Medical Care Survey (NAMCS) [11] in the United States, the Bettering the Evaluation and Care of Health (BEACH) study [12] in Australia, and the National primary Medical Care survey (NatMedCa) in New Zealand [13]. These have provided fine-grained cross-sectional data on the clinical content of general practice/primary care consultations and the clinical behaviours of GPs/primary care physicians. The longevity of two of these programs (BEACH 1998–2016; NAMCS 1973-present) has also provided important findings on temporal trends in general practice/family practice content and clinician clinical behaviour [14, 15]. The study methodologies, involving dedicated, structured contemporaneous recording of consultation data by the clinician participant for multiple unselected consecutive consultations, is more labour-intensive than extraction of data from pre-existing clinical electronic medical records (EMRs). However the methodology has considerable advantages in some areas, e.g., clinician focus for the dedicated recording period using a structured data recording format would be expected to produce more complete and reliable data [16], and there is capacity to explicitly link clinician’s in-consultation actions (prescribing, test-ordering, specialist referral etc.) to the problems/diagnoses which prompted them.

As well as BEACH, NAMCS and NatMedCa, there have been other small, more limited studies of GPs purposively recording details of consecutive consultations for research purposes in, for example, Canada [17] and the United States [18].

Reports of clinical encounters of registrars or trainees in general practice training (apart from reports from the ReCEnT study) are scarce. Despite the importance of the area both clinically and educationally, we are aware of only one peer-reviewed publication on the content and nature of patient encounters with registrars in Australian general practice training (a paper reporting comparisons between registrars and established GPs on six outcomes, without multivariable analyses) [19]. Internationally, a cross-sectional study of 74 Dutch general practice trainees (using EMR data) established the prevalence and characteristics (including patient problems/diagnoses) of GP trainees’ consultations [20] and compared these to consultations of trainee supervisors [21], but without multivariable analyses. An earlier English study compared the clinical in-consultation activities of 207 GP trainees with those of their supervisors, adjusted for some aspects of case mix (patient age and sex, and reason for consultation) [22]. There has also been a United States study of the differences in patient mix of 21 male and 20 female family medicine residents [23]. Comprehensive studies of registrar/trainee clinical experience and case-mix are, to our knowledge, otherwise lacking, though there are studies of limited aspects of experience/case mix (for example, 13 trainees’ audits of patients with rheumatological disease [24]) and very limited studies of overall case-mix (for example, a single registrar’s experience [25]).

The ReCEnT study has aimed (since 2010) to document longitudinally the consultation-based clinical and educational experiences of individual general practice registrars. Additionally, along with some other aims (discussed below) it seeks to establish the associations of registrars’ patterns of practice (the patients and conditions they see, and the in-consultation clinical and educational actions they pursue). An initial protocol has been published previously [26], but, while the basic study methodology has been retained, over subsequent years there have been iterations of aspects of the study and a major change in 2020 with a move from paper-based data recording to online data recording.

In this protocol we present the current methodology of the ReCEnT project. ReCEnT is first and foremost an educational program [27] (in the educational context, ReCEnT is a Patient Encounter Tracking and Learning tool (PETAL) [28]), but with a prominent research function [29]. The educational and research elements are closely integrated and substantively inform each other. This protocol is concerned with the research components of ReCEnT.

## Methods/design

### Study design

ReCEnT is an ongoing inception cohort study.

### Study aims

The study aims to document multiple variables from the key domains of the patient consultations of GP registrars: namely, registrar, practice, patient, and consultation variables (described further  'Data collection', below). The consultation variables are further categorized as consultation content and consultation actions variables. A further consideration is that consultation variables are also characterized as clinical and educational consultation variables.

This data is used to meet a range of specific aims and answer a range of research questions, as detailed below.

### Setting

Data is collected during registrars’ clinical consultations in community-based Australian general practices. The participating practices are teaching practices of the Australian General Practice Training (AGPT) program [30]. Data is collected only during office-based consultations. It is not collected during home visits or residential care visits. Data is not collected in Aboriginal Medical Services or Australian Defence facilities.

### Participants

Participants are registrars (trainees in specialist general practice vocational training) in the AGPT program undertaking their three mandatory general practice-based training terms, each of which represents six months full-time work. Participating registrars are in training with ReCEnT-participating RTOs/RTPs.

The current RTOs are GP Synergy (all registrars in the state of New South Wales and the Australian Capital Territory), Eastern Victoria General Practice Training (39% of all registrars in the state of Victoria [31]), and General Practice Training Tasmania (all registrars in the state of Tasmania). These RTOs train 43% of Australia’s GP registrars [31], with a combined intake of 900 registrars per year. This configuration of participating RTOs has been in place since 2016 when there was a major reorganization of general practice vocational training. Prior to this, five then-RTPs participated (with geographic footprints of the whole of Tasmania and regions within New South Wales, South Australia, Victoria, and Queensland). In 2023 the delivery of the AGPT program will move from the RTOs to the Royal Australian College of General Practitioners (RACGP) and the Australian College of Rural and Remote Medicine (ACRRM).

### Recruitment

Recruitment takes place upon registrars’ commencement of the first general practice-based training term of the AGPT program. Registrars enrolled with participating RTOs are required to collect project data as a routine part of their educational programs [29]. Registrars may choose to also provide informed consent to their ReCEnT data being used for research purposes.

Recruitment is carried out at the level of the participating RTO. The sample frames are the RTO-held lists of registrars in general practice training terms 1, 2 or 3. At inception, each registrar is assigned a unique ReCEnT project ID. The list linking ID and registrar is held by the registrar’s RTO.

Before each 6-monthly data collection period all Term 1 registrars in the participating RTOs receive a detailed live orientation session (either face-to-face or via video conference) delivered on a regional basis. Recordings of the orientation session are available online for registrars who may have missed it or who wish to review the orientation. A video illustrating the process of recording of data in the online platform is also provided, along with a ReCEnT data entry manual, and Frequently Asked Questions (FAQs) document.

### Data collected

Data for the core ReCEnT project is collected via three means.

- A registrar ‘Characteristics’ questionnaire completed by all Term 1 registrars prior to commencing consultation data recording in Term 1. This elicits demographic data for the registrar.
- A ‘Practice and Projects (PnP)’ questionnaire completed by all Term 1, 2 and 3 registrars prior to commencing consultation data recording for that term. This elicits data regarding the individual general practice in which the registrar practices/trains during that term.
- Contemporaneously recording data on individual Case Report Forms (CRFs) for individual patients after each of 60 consecutive consultations (in each of a registrar’s three general practice training terms).

The core items of the questionnaires have been subject to minor iterations during the course of the project (2010–2022). There is also provision for a small number of items to be added to the questionnaires or CRF for a single round (or finite number of rounds) of data collection. For example, items contributing to a discrete choice experiment around antibiotic prescribing were added to the PnP during the two data collection rounds in 2020, then removed.

From the questionnaires and CRFs data is collected on a range of core variables. For descriptive and practical analytical purposes these core variables are characterized as:

Registrar ‘Characteristics’ variables are: gender (female/male/another gender identity (optional: please specify), prefer not to say); date of birth; country of birth; Aboriginal person; Torres Strait Islander person; language mainly spoken at home; university qualification in a health-related field before qualification as a doctor (and which field); university qualification in a non-health-related field before qualification as a doctor (and which field); country of primary medical degree where qualified as a doctor; university granting primary medical degree; year of graduation as a doctor; years of full time equivalent experience in hospital (after internship) prior to entering general practice training; post-graduate qualifications in medicine (and which ones); general practice fellowship program (RACGP or ACRRM); training pathway (general or rural); capability to conduct consultations in a language other than English (and which ones).

Practice and Projects’ variables are: rurality of the practice location using the Australian Standard Geographical Classification-Remoteness Area (ASGC-RA) classification [32], socio-economic status of the practice location using the Socio-economic Indexes for Areas (SEIFA) Index of Relative Socio-economic Disadvantage [33], region of the associated training organisation, registrar worked at the practice in a previous training term; date training term at the practice commenced; training term level (1, 2, or 3); number of full-time-equivalent general practitioners at practice (< 2, 2–4, 5–9, 10 +); number of general practice sessions worked each week (session is equivalent to a morning or afternoon of work); register does other regular non-GP medical work (plus number of sessions per week and area of work in clinical, education or research); practice routinely bulk bills all patients or certain categories of patients (pensioner and healthcare concession card holders, children under 16 years old, other groups) (see below regards ‘bulk billing’); registrar participates in roster for providing care after normal working hours; registrar undertakes nursing home visits; registrar undertakes home visits; registrar provides refugee health services; participates in the local hospital Emergency Department roster; registrar has rights to admit patients to the local hospital; registrar attends regular practice clinical meetings and/or practice journal club; registrar provides teaching at the practice (to medical students, to other students and what type).

In Australia the term “bulk-billing” is used to describe a form of payment for medical services. The Australian Commonwealth government operates a national health insurer, Medicare, which provides payment for almost all medical services. Doctors can opt to accept the Medicare payment for the service they provide (called “bulk-billing”) or charge the patient a higher fee (termed “private billing” where the patient pays the higher fee in full and receives a rebate from Medicare for part of the fee).

CRF variables can be grouped as patient variables, consultation variables, consultation action variables.

Patient variables – age; gender; new patient at the practice; new patient to the registrar; identifying as an Aboriginal and/or Torres Strait Islander; having a non-English speaking background.

Consultation variables – date; duration; payment items, payment method; consultation conducted in a language other than English; problem(s)/diagnose(s); new problem/diagnosis for patient; the patient has been seen before for the problem/diagnosis.

Consultation action variables – observation(s), examination(s), medication(s) prescribed/given/recommended; medication(s) dose(s) reduced with the intention to deprescribe in the future; medication(s) deprescribed in the consultation; antibiotic prescription intended for immediate or delayed use; investigations ordered; imaging and other tests ordered; procedures undertake; referrals made; follow-up arranged; learning goals generated; and recourse to in-consultation advice/information/assistance (sources of information).

Most consultation action variables are ‘clinical’ variables but there are also ‘educational’ variables – recourse to in-consultation advice, information, or assistance (from various sources, including the supervisor), and generation of learning goals.

See Tables 1, 2 and 3 for further definition of the consultation, consultation action variables and the educational variables in the ReCEnT dataset.

**Table 1:** Data items collected for each consultation

Data Item	Description
Date of consultation	Calendar date
Duration of consultation	In minutes
Age	Recorded as months up to 23 months. Recorded as years for 2 and above
Gender	Female, male, or non-binary
Payment Items	Item numbers pertaining to the consultation as defined by the government health insurer (Medicare)
Payment method	Entity paying for the consultation, being one of: bulk-billed, private billed, insurer for workplace injury, other, no charge
New to practice	Yes/No
New to registrar	Yes/No
Aboriginal	Yes/No. “Yes” if the patient identifies as Aboriginal
Torres Strait Islander	Yes/No. “Yes” if the patient identifies as Torres Strait Islander
Non-English-speaking background	Yes/No. “Yes” if the patient does not speak English at home as their primary language
Consultation conducted in language other than English	The language spoken. This does not include use of a translator or simple greetings only
Problems/Diagnoses	The issues dealt with during the consultation. Up to 4 can be listed. If more than four were dealt with then the participant picks the four that they judge as most important
Problem status	New/Old. This is “New” if the patient has never seen a doctor before for this problem
Previously seen for this problem	Yes/No. “Yes” if the participant has seen the patient for this problem before

**Table 2:** Consultation action data items collected when applicable

Observations	Blood pressure, heart rate, height, weight, BMI, temperature, oxygen saturation, respiratory rate
Examinations	System(s) examined whether in full or in part and linked to which problem(s)
Medications prescribed	Any medication prescribed, given during the consultation, or recommended to be obtained and taken by the patient. Up to 8 can be listed. The following 4 items are collected for each medication
Drug name	Identification of the medication
Administration route	How the medication is taken or applied to the patient
Drug status	New/Continued. “New” if the patient was not taking this medication before the consultation
Linked to problem	List the problem number(s) this medication related to
Medications reduced with the intention to cease later	Any medication that is intended to be deprescribed but requires weaning to be able to cease safely. Up to 8 can be listed. The following 5 items are collected for each medication
Drug name	Identification of the medication
Administration route	How the medication is taken or applied to the patient
Prescription duration in months	Indicate if the patient has been taking the medication for less than 3 months or for 3 months or more
Reason(s) to reduce medication	List one or more reasons
Linked to problem	List the problem number(s) this medication related to
Medications ceased	Any medication that is deprescribed. Up to 8 can be listed. The following 5 items are collected for each medication
Drug name	Identification of the medication
Administration route	How the medication is taken or applied to the patient
Prescription duration in months	Indicate if the patient has been taking the medication for less than 3 months or for 3 months or more
Reason(s) to cease medication	List one or more reasons
Linked to problem	List the problem number(s) this medication related to
Pathology	List up to 12 tests requested and indicate which problems/diagnoses each one related to
Referred imaging / other tests	List the name of the imaging or other test, the body site it related to and indicate which problems/diagnoses each one related to
Procedures	List the procedures undertaken in the consultation and indicate which problems/diagnoses each one related to
Referral	Record any referrals to a government funded clinic/specialist, private specialist, private allied health, other agency, emergency department or hospital. Record the specific type of service and indicate which problems/diagnoses each one related to
Follow-up arranged	When specific follow-up arrangements are made, record if it is an appointment with the participant, another GP at the same practice, the practice nurse, or via telephone
Antibiotic prescribing	When an antibiotic was prescribed indicate if it was for immediate use or was a delayed prescription

**Table 3:** Educational data items collected for each consultation

Learning goals	Indicate which problems/diagnoses the participants wanted to look up further information about after the consultation was completed
Sources of information	Indicate which problems/diagnoses assistance was sought for during the consultation. Indicate where the information was sourced (supervisor or other doctor in the practice, a specialist and what type, other health professional and what type, book and which one, electronic resource and which one, any other and what type) and whether it was to assist with diagnosis, management or both

### Data collection instruments

Prior to 2020, these questionnaires (‘Characteristics’ and ‘PnP’) and CRFs were distributed and completed in hardcopy format. They are now completed via an online platform. See Additional materials for examples of the CRF (Additional file 1), questionnaires (Additional files 2 and 3).

The data is currently collected using an online platform (ReCEnT Online) (since 2020 for the CRF and 2021 for the questionnaires). On first access to ReCEnT Online, participating registrar data is collected with the first ReCEnT questionnaire. With each GP training term further data (mainly data related to the registrar’s current training practice) is collected via the PnP questionnaires. Each term these questionnaires are completed prior to the in-consultation data collection for 60 consecutive consultations being commenced using the online CRFs.

The ReCEnT Online interface is a web-based application using ASP.NET, HTTPS protocol, and the minimum browser requirement security support is TLS1.2. Only authenticated users are allowed access to the interface, whose business logic is implemented using.NET Framework. The data repository is SQL Server 2016 and all files, components and data are hosted on a Microsoft Azure cloud service.

### Data collection

Data collection is timed to coincide with approximately the mid-point of the participant’s GP training term. The 60 consultations represent approximately a week of clinical consultations for a full-time registrar.

Registrars record data for 60 consecutive office-based consultations. Consultations excluded from data collection are:

- Home visits and nursing home (aged care facility) visit consultations
- Consultations conducted as part of a ‘single purpose’ clinic. These include vaccination clinics and cervical screening clinics.

The rationale for these exclusions is:

For home visits and nursing home visits, we do not have the technological capacity to accept data entry from mobile devices. Prior to 2020, we considered that taking CRF pads to nursing homes and patients’ homes entailed too great a risk of misplacing and losing the pads.

For ‘single purpose’ clinics, this reflects the ReCEnT project being first and foremost an educational program. While having data on these clinics would be useful from a research/epidemiological viewpoint [34], it could compromise the educational element. The primary educational function of ReCEnT is in registrars’ data being processed and returned to the registrar as a reflective report [27, 29]. A registrar report containing, for example, a large proportion of data from brief consultations conducted as part of an influenza or Covid-19 vaccination clinic would be a poor substrate for the registrar’s reflection on practice.

### Data coding

Problems/diagnoses, pathology ordered, imaging ordered, and referrals are coded according to the International Classification of Primary Care, 2nd edition-Plus (ICPC-2Plus.) [35] Medications are coded according to the Anatomic Therapeutic Chemical (ATC) Classification [36]. Procedures are coded according to a list of procedures appropriate to general practice training compiled via a Delphi process [37].

### Data analysis

Throughout the course of ReCEnT, multivariable analyses have been conducted by the Clinical Research Design and Statistical Services unit of the Hunter Medical Research Institute [38] which works in partnership with the University of Newcastle, Australia. Advantages of this collaboration are continuity of individual statistician work on the project and consistency of statistical approaches to our various types of research questions.

A range of approaches have been used, and are anticipated to be used, in analyses of ReCEnT data. The variables collected have been used/may be used, in different analyses, as either outcome or independent variables.

The statistical approaches used have been/will be determined by the type of research question being answered.

## Discussion

Strengths of the study are the granular detail of clinical practice relating to patient demographics, presenting problems/diagnoses, medication decisions, investigations requested, referrals made, procedures undertaken, follow-up arranged, learning goals generated, and in-consultation help sought; the linking of the above variables to the presenting problems/diagnoses to which they pertain; and a very high response rate. The study is limited by not having information regarding severity of illness, medical history of the patient, full medication regimens, or patient compliance to clinical decisions made at the consultation.

We categorize the types of research questions we attempt to answer as:

### ‘Mapping’ analyses

Prior to the ReCEnT project, the content of Australian GP registrars’ consultations was unknown, and there was very limited international evidence for the content of the ‘black box’ [20] of general practice trainees’ consultations.

With ReCEnT, we have attempted to open that black box and ‘map’ what GP registrars see and do. This involves documenting the overall composition of registrars’ patient population by demographics and disease classification of the patients’ problems/diagnoses [39].

It also involves establishing the prevalence of particular conditions (problems/diagnoses) or patient demographics as a proportion of all registrar problems/diagnoses. This is typically expressed as proportions and 95% confidence intervals, and often interpreted in the context of similar findings from the practice of established GPs (usually with findings from the BEACH study). For example, the proportion of registrar patients aged 65 years and over is less than that of established GPs [40].

We are also able to ‘map’ registrars’ clinical behaviours (prescribing (and deprescribing), test-ordering, referrals, and organization of follow-up). For example, the prevalence of long-acting reversible contraception prescribing by Australian GP registrars in the ReCEnT study is higher than has been previously estimated in established GPs [41].

### Exploratory analyses

Exploratory analyses allow us to establish associations of a wide range of registrar clinical exposures—for example, patient demographics (older patients [40], Aboriginal and Torres Strait Islander patients) [42] and problems/diagnoses (for example, atopic dermatitis [43], post-natal care [44], vertigo [45]); and registrar actions (prescribing [46], deprescribing [47], delayed prescribing [48], test-ordering [49], referral [50], and preventive activities [51]). We can also examine the associations of structural aspects of the training experience. For example, the associations of training in rural and remote location [52], or of contributing to the practice after-hours roster [53]. For these analyses we typically use multivariable regression models, as appropriate to the outcome variable, e.g., linear, logistic or negative binomial regression for continuous, binary or count responses, respectively. We conduct the regressions within the generalised estimating equations (GEE) framework to account for repeated measures within GP-registrars. We generally use GEE rather than mixed models to account for non-independence due to repeated measures, since our interest is in effect estimates averaged across registrars, rather than registrar-specific effects (as produced with mixed (random effects) models). To estimate the GEE, we typically assume an exchangeable working correlation structure.

### Mapping and exploratory analyses of registrars’ educational actions

While all our analyses are of relevance to general practice vocational education and training, some analyses are of specifically educational topics. For example, the sources of in-consultation information, advice, and assistance that registrars access [54], and the learning goals registrars generate during the consultation to be pursued post-consultation [55]. Multivariable regression methods are mainly used in these analyses.

### Mapping and exploratory analyses of other outcomes

Analyses can also be performed to explore prevalence and associations of somewhat more complex constructs – for example, continuity of care [56] and doctor-patient gender concordance [57] in registrars’ practice. Multivariable regression methods are mainly used in these analyses.

### Longitudinal ‘within-registrar’ analyses

In the Australian specialist GP training program, registrars complete three 6-month (full-time equivalent) terms in clinical general practice. ReCEnT data collection occurs at approximately the midpoint of each of these three terms. We can thus use this data to examine temporal changes (from Term 1 to Term 2 to Term 3) in various outcomes at the individual registrar level. For example, a past analysis analysed within-registrar changes in benzodiazepine prescribing [58].

The statistical approaches used for ‘within-registrar’ longitudinal analyses utilise only within-registrar information, by treating registrar as a fixed effect in multivariable regression models. For example, in the previous analysis of benzodiazepine prescribing trends, conditional (fixed-effects) logistic regression was used to estimate the association between successive training Terms and the odds of benzodiazepine prescribing within registrars.

### Longitudinal ‘within-program’ analyses

As well as examining changes in individual registrars’ clinical experiences or actions as they progress through training, we can examine temporal trends in registrars’ clinical experiences or actions at the program level. These analyses estimate the effect of time as a combination of within-registrar and between-registrar effects, with temporal trends quantified either as a function of calendar time (since 2010), or training term. In these analyses, the effect of time – allowing for repeated measures within registrars – has been estimated both using generalised linear mixed models (GLMM; treating registrar as a random effect) and generalised estimating equations (GEE). In both the GLMM and GEE frameworks, the response variable distribution is defined as appropriate to the outcome. For example, we have demonstrated a program-level decrease over time (calendar year) in prescribing of benzodiazepines [58], and in antibiotic prescribing for a number of non-pneumonia acute respiratory tract infections, using mixed effects logistic regression models. We have also examined trends in pathology test-ordering by registrars [59], over successive training terms. Here we have used negative binomial models and zero-inflated negative binomial models, within the GEE framework to estimate the association between successive training terms and rates of pathology test ordering.

### Triangulation of ‘within-registrar’ and ‘within-program’ findings

Triangulating findings of these two analytical approaches has the potential to provide insights into how registrar learning and registrars’ adoption of clinical behaviours operate within the apprenticeship-like model of GP vocational practice.

The findings of individual registrars’ benzodiazepine and antibiotic prescribing for acute bronchitis and acute upper respiratory tract infections not changing from term to term, but overall prescribing decreasing from year to year, may indicate that registrars’ clinical behaviours in general may be more influenced by the practice environment (for example, the ‘prescribing culture’) than by specific education (note, though, the findings of some intensive targeted ‘education interventional’ analyses, below). The context of our findings for the reduction in within-program registrar antibiotic prescribing is that this is consistent with reductions in overall antibiotic prescribing in the wider Australian GP population [60].

### Testing efficacy of educational interventions

ReCEnT data, being recorded regularly every six-months, can be used to assess the efficacy of educational interventions timed to occur between data collection rounds. Of necessity, this does not entail randomized controlled trials (RCTs). These are impractical in the setting of Australian general practice vocational training. Educational sessions are conducted at the level of RTO, or regional level within RTO, but cluster RCTs are not practicable due to the large size of the educational clusters and, especially, the fact that educational considerations outweigh research imperatives. Randomizing registrars to differing educational content is not considered educationally appropriate. Instead, our approach is to use a non-equivalent control groups design. ‘Educational innovations’ (rather than true ‘interventions’) in the form of new educational sessions/packages are scheduled to occur between ReCEnT data collection rounds. The educational sessions/packages become part of the ongoing RTO educational program. This process has been followed for antibiotic prescribing for URTI and acute bronchitis [61], opioid prescribing [62], pathology test-ordering, benzodiazepine prescribing, and deprescribing of inappropriate medicines.

Our analyses in these evaluations have been based on an ‘Intention-to-educate’ principle and utilised logistic regression (given a binary response) within a GEE framework, adjusted for relevant independent variables. The key predictors of interest have been time (post vs pre intervention); treatment group (intervention vs control); and an interaction term between time and treatment group. The estimand of interest has been the difference between intervention and control groups in the pre to post change in the odds of prescribing, with the P-value associated with the interaction term used to assess statistical significance of the group difference.

While we are unable to conduct randomized studies of the educational innovations, the large number of potential confounding independent variables measured in ReCEnT allows for fine-grained adjustment in our modelling.

These analyses are the final step in an iterative educational model whereby ReCEnT data is used initially to identify a need for educational action in registrars’ practice (and to quantify the problem to help prioritize the importance of the topic for inclusion in crowded educational programs). The ReCEnT-established associations of the target behaviour (that is, of the outcome in the relevant ReCEnT analyses) help inform the content of the educational innovation. ReCEnT data is then used to assess the efficacy of the educational innovation in changing registrar behaviour.

A further aspect is to triangulate this finding of change in registrars’ behaviour with change in registrars’ knowledge or attitude (assessed via concurrent pre- and post-session questionnaires) with the same innovation. For example, for antibiotic prescribing [61, 63] or opioid prescribing [62, 64] educational innovations. A conclusion from these comparisons is that changes in knowledge and/or attitude may not be reflected in change in clinical practice. A consequence is that the educational innovations we now design, informed by ReCEnT data, consider aspects beyond instilling knowledge. These educational sessions/packages also address other areas of Michie’s behaviour change model [65].

### Combination of ReCEnT data with data from other sources

There is potential to answer research questions utilizing ReCEnT data in combination with other data sources.

Currently, we are using ReCEnT data to compile an index of low-value or inappropriate clinical activities (the QUestionable In-Training Clinical Activities, QUIT-CA index) [66]. Combining this data with data collected by GP Synergy as part of registrars’ formative assessment program (observed in-consultation performance [67] and in-training formative examinations) along with data on registrars’ summative examination (RACGP Fellowship examination) performance, we will be able to explore the relationship of unobserved (‘actual’) clinical performance with observed clinical performance and with formative and summative examination performance.

We are also collecting data on patients’ evaluation of the patient-centredness of GP Synergy registrars’ consultations using the Consultation And Relational Empathy (CARE) measure [68]. We are planning to examine CARE scores (as an outcome), including exploring associations of registrars’ CARE scores. We also anticipate that CARE scores will be of utility (as a measure of registrars’ patient-centredness) as an independent variable in ReCEnT analyses with a range of outcomes.

The study enables identification of training needs and translation of subsequent evidence-based educational innovations into specialist training of general practitioners.

## Supplementary Information

**Additional file 1.** Registrar Clinical Encounters in Training (ReCEnT) Online Encounter Form Description of Data: Copy of the online encounter form registrars complete for individual patients after each of 60 consecutive consultations (in each of a registrar’s three general practice training terms). This is the online version of the Case Report Form and elicits data on patient variables, consultation variables, and consultation action variables. 

**Additional file 2.**  Registrar Clinical Encounters in Training (ReCEnT) Registrar Characteristics Questionnaire Description of Data: Copy of the registrar ‘Characteristics’ questionnaire completed by all Term 1 registrars prior to commencing consultation data recording in Term 1. This elicits demographic data for the registrar.

**Additional file 3.** Registrar Clinical Encounters in Training (ReCEnT) Practice and Projects (PnP) Questionnaire Description of Data: Copy of the registrar ‘Practice and Projects (PnP)’ questionnaire completed by all Term 1, 2 and 3 registrars prior to commencing consultation data recording for that term. This elicits data regarding the individual general practice in which the registrar practices/trains during that term.

## Data Availability

Not applicable. The responsible ethics committee has advised that this must not be made available as some participants in earlier stages of the study have not given explicit consent for their data to be shared.

## Abbreviations

ACRRM: Australian College of Rural and Remote Medicine
AGPT: Australian General Practice Training
ASGC-RA: Australian Standard Geographical Classification-Remoteness Area
ATC: Anatomic Therapeutic Chemical
BEACH: Bettering the Evaluation and Care of Health
CARE: Consultation And Relational Empathy
CRF: Case Report Forms
EMR: Electronic Medical Record
FAQ: Frequently Asked Questions
GEE: Generalised Estimating Equation
GLMM: Generalised Linear Mixed Model
GP: General practitioner
ICPC-2Plus: International Classification of Primary Care, 2nd edition-Plus
NAMCS: Primary Care Network Survey and the National Ambulatory Medical Care Survey
NatMedCa: National primary Medical Care
PETAL: Patient Encounter Tracking and Learning
PnP: Practice and Projects
QUIT-CA: QUestionable In-Training Clinical Activities
RACGP: Royal Australian College of General Practitioners
RCT: Randomised Clinical Trial
ReCEnT: Registrars Clinical Encounters in Training
RTO: Regional Training Organisation
RTP: Regional Training Providers
SEIFA: Socio-economic Indexes for Areas
