# Supplementary material for: The Registrar Clinical Encounters in Training (ReCEnT) cohort study: updated protocol
Source: BMC Prim Care. 2022 Dec 16;23:328. doi: 10.1186/s12875-022-01920-7 (PMC9755776; doi:10.1186/s12875-022-01920-7)
Supplement: Supplementary file 1 — Additional file 1. Registrar Clinical Encounters in Training (ReCEnT) Online Encounter Form Description of Data: Copy of the online encounter form registrars complete for individual patients after each of 60 consecutive consultations (in each of a registrar’s three general practice training terms). This is the online version of the Case Report Form and elicits data on patient variables, consultation variables, and consultation action variables. [file 12875_2022_1920_MOESM1_ESM.pdf]

Registrar Clinical Encounter in Training - Encounter 14 / 60

Encounter details

Encounter number: 14

Billing *: Private | Bulk bill | Workers comp | No charge | Other

Date *: 22/02/2022

Medicare (MBS) Items *
Maximum 4 items

Duration *
Maximum 120 minutes

Patient details

Age *: Years * | Months *

Gender *: Male | Female | Non Binary

New to practice *: Yes | No

Aboriginal: Yes | No

New to me *: Yes | No

Torres Strait Islander: Yes | No

Non English speaking background: Yes | No

Consulted in a language other than English

Problem/Prov Diagnosis 1

Description *

Status *: Old | New

Seen by you *for this problem* ever before? *: Yes | No

Add more

Medication(s) prescribed

Linked to problem(s): 1

Drug name

Drug status: New | Continued

Administration route

Add more

Medication(s) reduced with intention to cease later

Linked to problem(s): 1

Drug name

Administration route

Prescription duration in months: <3 months | >3 months

Reason(s) to reduce medication: Lack of efficacy | Non compliance | Drug interactions | No longer indicated | Side effects | Other

Add more

Medication(s) ceased

Linked to problem(s): 1

Drug name

Administration route

Prescription duration in months: <3 months | >3 months

Reason(s) for cessation: Lack of efficacy | Non compliance | Drug interactions | No longer indicated | Side effects | Other

Add more

Pathology ordered

Test name

Linked to problem(s): 1

Add more

Referred imaging / other tests

Test name

Body site

Linked to problem(s): 1

Add more

Procedures performed

Procedure name

Linked to problem(s): 1

Add more

Referrals made

Public clinic / specialist — For problem(s): 1 — Specify

Private specialist — For problem(s): 1 — Specify

Private allied health — For problem(s): 1 — Specify

Other agency — For problem(s): 1 — Specify

ED / hospital — For problem(s): 1 — Specify

Scheduled follow-ups

GP appointment with you — For problem(s): 1

GP appointment with another Dr at the practice — For problem(s): 1

Practice nurse appointment — For problem(s): 1

Telephone follow-up — For problem(s): 1

Generated learning goals

Linked to problem(s): 1

Sources of information for patient care during consultation

Supervisor/other Dr in practice — For problem(s): 1 — Diagnosis | Management

Specialist — For problem(s): 1 — Diagnosis | Management

Other health professional — For problem(s): 1 — Diagnosis | Management — Specify

Books — For problem(s): 1 — Diagnosis | Management — Specify

Electronic resources — For problem(s): 1 — Diagnosis | Management — Specify

Others — For problem(s): 1 — Diagnosis | Management — Specify

Antibiotics prescribed

If you prescribed or recommended an antibiotic, was it: For immediate use | Provided as a script to be filled later | Arranged for patient to collect script later

Reset | Save | Submit
