# Supplementary material for: The Registrar Clinical Encounters in Training (ReCEnT) cohort study: updated protocol
Source: BMC Prim Care. 2022 Dec 16;23:328. doi: 10.1186/s12875-022-01920-7 (PMC9755776; doi:10.1186/s12875-022-01920-7)
Supplement: Supplementary file 2 — Additional file 2. Registrar Clinical Encounters in Training (ReCEnT) Registrar Characteristics Questionnaire Description of Data: Copy of the registrar ‘Characteristics’ questionnaire completed by all Term 1 registrars prior to commencing consultation data recording in Term 1. This elicits demographic data for the registrar. [file 12875_2022_1920_MOESM2_ESM.pdf]

Welcome to ReCEnT Online

The ReCEnT project aims to document clinical and education content of your consultations with patients, and provide you with a report to enable you to reflect upon this.

Completing ReCEnT is a compulsory part of your training program, to be completed in your first, second and third GP terms.

You are required to:

- Complete a questionnaire about your characteristics
- Complete a questionnaire about your CURRENT practice's characteristics (plus a few other questions)
- Read the Information Statement and consent to your data being used for research
- Record details of 60 CONSECUTIVE consultations. Only record consultations conducted in the general practice office setting (i.e. DO NOT record consultations conducted in a nursing home, or on a home visit). As well, do not record consultations as part of a specialised clinic e.g. immunisation, flu-needle clinic, or Women's Health clinic.

Please begin by completing the form(s) below:

Characteristics
Practice and projects
Consent

Personal Details

Gender

Male | Female | Another gender identity | Prefer not to say

Optional: please specify other gender identity

Date of Birth (DD/MM/YYYY) *

Country of Birth *

Do you identify as Aboriginal? *

Yes | No

Do you identify as Torres Strait Islander? *

Yes | No

Which language do you mainly speak at home? *

English | Other

Please specify

Qualifications

Did you obtain university qualifications in a *health-related* field **BEFORE** you qualified as a doctor? *

Yes | No

Please specify the field/s

Ambulance officer/ Paramedic	Dentistry
Dietetics	Nursing
Occupational Therapy	Pharmacy
Physiotherapy	Psychology
Social Work	Speech Pathology
Other	

Please specify (Other)

Did you obtain university qualifications in a *non health-related* field **BEFORE** you qualified as a doctor? *

Yes | No

Please specify the field/s

Where did you qualify as a doctor (primary medical degree)? *

Australia | Other

Please specify the country

At which University did you obtain your primary medical degree? *

What year did you graduate as a doctor? *

How many (full-time-equivalent) years have you worked in a hospital **post-internship or post-AMC qualification** prior to entering General Practice Training? Answer to the nearest year. *

Have you obtained any post-graduate qualifications in medicine e.g. MPH, DipRACOG? *

Yes | No

Please specify which qualifications

Which College are you working towards Fellowship with? (select both if applicable) *

RACGP | ACRRM

Which pathway are you enrolled in? *

General | Rural

Do you think you have capacity to conduct a consultation in a language/s other than English?

Yes | No

If yes please specify other language(s) *

Save draft | Next
