# Supplementary material for: The Registrar Clinical Encounters in Training (ReCEnT) cohort study: updated protocol
Source: BMC Prim Care. 2022 Dec 16;23:328. doi: 10.1186/s12875-022-01920-7 (PMC9755776; doi:10.1186/s12875-022-01920-7)
Supplement: Supplementary file 3 — Additional file 3. Registrar Clinical Encounters in Training (ReCEnT) Practice and Projects (PnP) Questionnaire Description of Data: Copy of the registrar ‘Practice and Projects (PnP)’ questionnaire completed by all Term 1, 2 and 3 registrars prior to commencing consultation data recording for that term. This elicits data regarding the individual general practice in which the registrar practices/trains during that term. [file 12875_2022_1920_MOESM3_ESM.pdf]

Training details

Have you worked in your current practice previously during your training? *

Yes | No

What date did you start your current placement (DD/MM/YYYY)? *

Which training term are you doing now? *

Term 1 | Term 2 | Term 3 | Extension

How many GPs (full time equivalents) work with you at this practice? *

<2 | 2-4 | 5-9 | 10+ | NA

How many general practice sessions do you work each week on average? *(n.b. 1 session = approx. 4 hours e.g. a morning session)* *

Do you do other regular non-GP medical work *

Yes | No

How many sessions do you do this other medical work on average each week?

Does this other medical work involve (select all that apply):

Clinical | Education | Research

Does your practice routinely bulk bill ALL patients? *

Yes | No

Which groups are bulk billed?

All pensioner/healthcare card holders | All children <16 years | Selected other patient groups | No patients are bulk-billed

Please specify which groups

Clinical extra hours

Does your clinical GP work at your current practice involve the following (select all that apply)? *

Rostered after hours care | Nursing home visits | Home visits | Refugee health | None of these options

Do you contribute to your local Emergency Department after hours roster / care? *

Yes | No

Do you have VMO rights and admit patients to the local hospital? *

Yes | No

Do you attend regular practice clinical meetings and/or journal club? *

Yes | No

Does your current clinical GP registrar role involve you teaching in your practice? *

Yes | No

Please specify *

Medical student | Other

Please specify (e.g. nursing students)

Billing

The education I received from my RTO prepared me for billing patients in general practice *

Strongly agree | Agree | Neutral | Disagree | Strongly disagree

How would you rate your understanding of billing processes in general practice (including the application of the range of item numbers relevant to general practitioners)? *

Very good | Good | Neither good nor poor | Poor | Very poor

How would you rate your confidence in using telehealth in general practice? *

Very confident | Somewhat confident | Neutral | Somewhat unconfident | Very unconfident

How concerned are you about patient confidentiality when using telehealth? *

Very concerned | Somewhat concerned | Neutral | Somewhat unconcerned | Very unconcerned

Save draft | Next
